# Static and Fatigue Behaviour of Double-Lap Adhesive Joints and Notched Metal Samples Reinforced by Composite Overlays

**DOI:** 10.3390/ma15093233

**Published:** 2022-04-29

**Authors:** Paweł J. Romanowicz, Bogdan Szybiński, Mateusz Wygoda

**Affiliations:** 1 Department of Machine Design and Composite Structures, Faculty of Mechanical Engineering, Cracow University of Technology, ul. Warszawska 24, 31-155 Cracow, Poland; bogdan.szybinski@pk.edu.pl; 2 Department of Product Technology and Ecology, College of Management and Quality Sciences, Cracow University of Economics, 31-510 Cracow, Poland; mateusz.wygoda@uek.krakow.pl

**Keywords:** adhesive joints, reinforcements, notches, static and fatigue tensile tests, double-lap joint, digital image correlation, finite element modelling, metal and composite structures

## Abstract

The use of composite overlays to increase the fatigue life of notched steel samples is discussed in this paper. For such purposes, in the first set of studies, static and fatigue tests as well as the detailed analytical and numerical analyses for samples with double-lap joints were performed. Based on such studies, the shapes of the composite overlays were set. For a better understanding of the failure forms of the investigated adhesive joints, the experimental studies were monitored with the use of digital image correlation. In the second set of experimental studies, the static and fatigue tensile tests were performed for steel samples with a rectangular opening with rounded corners reinforced by composite overlays. The different shapes (square 45 × 45 mm and long stripes 180 × 15 mm) and composite materials (GFRP and CFRP) were used as overlays. The obtained improvement of fatigue life was in the range of 180–270% in the case of the rectangular overlays and 710% in the case of application of the overlays in the form of the long stripes. This was also confirmed by numerical analyses in which a reduction in the stress concentration factor from 2.508 (bare sample) through 2.014–2.183 (square 45 × 45 mm overlays) to 1.366 (overlays in the form of long stripes 180 × 15 mm) was observed.

## 1. Introduction

It is well-established knowledge that the presence of various shape discontinuities [1,2], voids, cracks [3], holes [4,5], cracks around circular notches [6,7] or edge-notches [8] in structural elements substantially reduces their static [9,10] and fatigue performance [11,12,13]. The zones with such discontinuities are known as notches, and the accompanying disturbance of strain and stress distributions around the notches results in a local rise of stresses and strains [14]. This is a serious danger and may be the source of premature failure [14]. Assessment of this threat is well recognized; hundreds of papers studying this phenomenon have been published and the respective code design rules for structures with notches have been used since the beginning of the twentieth century [15]. In order to minimize the influence of the stress raisers on a lifetime of machines or structures, two general approaches have been accommodated. The first one relies on redesigning of structures or machine elements in order to reduce or omit the stress concentration [10]. The second technique relies on the introduction of local reinforcements [4,5,7] which spread around the close-notch neighbourhood [16]. This is justified both by analytical solutions [17] and experimental results [15], which confirm that the stress concentration affects only a limited zone around the opening, undercut, groove, crack, etc. Such a reinforcing technique has a long tradition and is still efficiently applied for distant, isolated notches [18]. The more versatile solution is the application of non-metallic overlays, which are adhesively bonded around the notches or cracks. In this case, not only the metallic but also concrete or composite and other structural elements can be recovered. The only limit in this case is the adhesiveness of joined surfaces. The very rapid development of material technology, mainly glass- and carbon-fiber-reinforced plastics (GFRP and CFRP, respectively) manufacturing, accompanied by the progress in bonding techniques, has offered new possibilities in reinforcement or repair techniques. In recent years, a series of papers related to the mechanical performance of various combinations of materials applied in joints between aluminium–CFRP [5], steel–CFRP [19,20,21], polymer-matrix composites [22], steel bridges–CFRP [23], concrete beams–CFRP [24], and welded beam-column connection–CFRP [25] were published. The general conclusions and observations for such combined structures say that it is possible to recover the fatigue resistance or mechanical endurance of the investigated structures or elements with faults or notches, but one must be aware of the presence of several obstacles to overcome to obtain the optimal structure.

The crucial recommendation for designing of adhesive joints is that the load should be transferred through the in-plane shear. This observation restricts the number of recommended shapes for bonded joints. The review of typical joints for structural applications is given in [26]. Many papers devoted to the mechanical durability of joints consisting of parts made from steel [27], CFRP [28,29,30], GFRP [31] and Honeycomb composites [32,33] can be quoted. Not only the material choice, but also the influence of the adhesive used for joints has been profoundly studied, showing that the improper choice of adhesive type may significantly reduce the strength of the joint [34].

In the case of joining different materials together, additional problems not appearing in monolithic structures are observed in experimental investigations of adhesive joints. Even though well-developed non-destructive inspection techniques are now available, destructive tests are still the main source of knowledge in the case of adhesive joint endurance assessment. Furthermore, the problem with the load transfer between joined elements appears along the edges of the bonds, and here various shapes of the joint edges are studied in numerous papers [13,35]. Most of the studies are concerned with the experimental bending of steel beams [2,25], concrete beams [24], open-hole steel plates [4], steel girders [19], and flat steel plates [20], and static [30,31,34] and fatigue tests of cracked steel plates [3,7,8], open-hole steel plates [4,6], aluminium plates with holes [5] and polymer-matrix composites [22]. What is more, Bocciarelli et al. [36] observed that the fatigue strength of the adhesive joint between steel and CFRP plates is higher than for welded connection. Numerous results of experimental fatigue strength studies are related to samples with circular notches [4,5] or with initial cracks [3,7,8].

Additional difficulty in the analysis of adhesive bonded joints is concerned with the negative influence of environmental conditions [32,37] such as low and high temperature [31,37,38], level of humidity [34,38], exposure to sunlight, presence of solvents, and aggressive environment, which may be the source of degradation processes.

The analytical assessment of stress and strain distribution in bonded joints uses different models, starting from the simplest average shear stress model and moving towards the two well-established approaches, namely the shear-lag Volkersen model [39] and the Goland–Reissner (G–R) concept [40]. The common feature of both models is the assumption that the stress is constant across the thickness of the adhesive, which is one of their deficiencies. The main advantage of the G–R model is the possibility of the calculation of the peel stress distribution, which is one of the failure modes of bonded joints observed in practice. Different modifications of the G–R model have been developed and can be found in [41,42,43,44]. Recently, another analytical model based on the stiffness matrix theory of framed structures was proposed by Areiza-Hurtado et al. [45]. The main deficiency of all mentioned models is their limitation to the linear elastic behaviour of the joining parts and adhesive materials. Such an approach is usually the source of overestimation of the results, which is not observed in the case of contemporary adhesives, due to the fact that their failure is affected by the elastic non-linear behaviour of the adhesives. This limitation was omitted by the model with material inelasticity proposed by Hart-Smith [46,47].

The effectiveness of the adhesive joint is limited not only by the materials used for core and overlays and the kind of the applied glue, but also by the adherent thickness. If the adherents become thicker than a certain limiting value, the joint loses its effectiveness. In addition, proper choice of relations between the rigidities of the basic material and reinforcing overlays appears to be crucial for joint endurance [9,37,41]. This requires an application of overlays with balanced stiffness [28,35] and optimal spew fillet geometry at the ends of the overlays to reduce the stress concentration in the adhesive [35]. The final decision concerning the type of the used adhesive and reinforcement is set after the complex analysis, which takes into account aspects such as: total cost, the durability of the joint, ease of maintenance, environmental impact, etc. All these aspects are included in the well-established life cycle assessment procedure [48].

The previous author’s investigations [9] revealed that the application of the composite overlays around the rectangular notches increases the static strength of the structure. It should be noted that S&P Resin 220 Epoxy Adhesive used for preparation of the adhesive joints was not applied for reinforcing of notched metal structures. The authors also did not find any information about reinforcing of steel structures with rectangular holes in the literature. Moreover, only limited information exists about the influence of overlay material and its shape on the fatigue strength. Because of this, the objective of the performed investigations was to confirm that the application of the reinforcements in the form of adhesively bonded joints—placed around the rectangular notches—also significantly improves the fatigue endurance of the highly loaded (above the yield limit at the notch) structural elements. For that purpose, a series of static and fatigue tests for steel bare notched samples and notched samples reinforced by composite overlays were performed. Additionally, broad tests for the assessment of fatigue properties of the used adhesive were conducted via the use of double-lap joint specimens. On the base of the obtained results, the program of fatigue investigation for reinforced samples with notches was set. The effectiveness of the proposed approach was proven by the increase in the fatigue life (more than seven times) in comparison with the non-reinforced specimen.

The paper consists of five sections. The introduction to the studied problem and the literature review are given in Section 1. The description of materials, samples, analytical solution, and finite element models are described in Section 2. The results of the static and fatigue experimental tests and analytical and numerical analyses for double-lap joints are presented and discussed in Section 3.1. The results of the fatigue tests for notched steel samples reinforced by composite overlays are shown in Section 3.2. Discussion of the presented study is provided in Section 4. The experimental tests and discussion are focused on the determination of the fatigue strength of the steel–steel and steel–composite adhesive joints with the use of S&P Resin 220 Epoxy Adhesive and evaluation of the possibilities of reinforcing of notched structures by different composite materials. The conclusions are given in Section 5.

## 2. Materials and Methods

### 2.1. Materials and Samples

**Figure 1:**
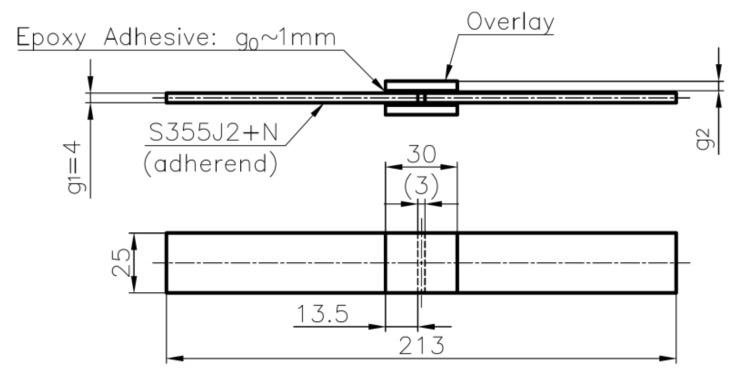
Geometry of samples with double-lap joint (samples no. 1–15).

**Figure 2:**
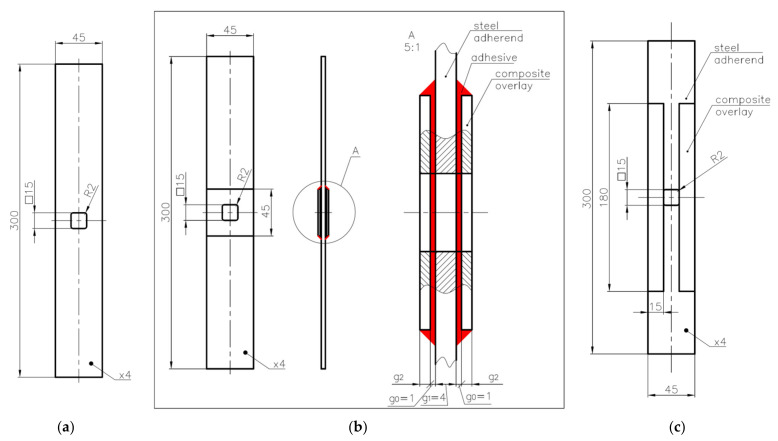
Geometry of tested samples with square notches with rounded corners: (**a**) without composite overlays—samples 16 and 17; (**b**) with two 45 × 45 mm composite overlays with hole at centre on both sides—samples 18–21; (**c**) with four rectangular 15 × 180 composite overlays on both sides—sample no. 22.

The study is focused on the analyses of static and fatigue strength of adhesive joints. Two kinds of bonded joints subjected to static and fatigue tension were tested in the study:

- static and fatigue strength of the double-lap steel–steel and steel–composite shear adhesive joints (geometry is given in Figure 1),
- static and fatigue strength of the notched metal samples reinforced by composite overlays (geometries are given in Figure 2).

The main parts (adherends) of the samples were made from S355J2+N steel with a thickness *g*_1_ = 4 mm. The chemical composition and mechanical properties of this steel are given in Table 1 and Table 2, respectively. Different kinds of materials were applied for reinforcing overlays of the investigated samples. These were made from the following materials:

- steel S355J2+N (U.S. Steel Košice, Košice, Slovak Republic),
- R-glass/epoxy composite HEXCEL TVR 380 with stacking sequence [+45°/−45°]_4_ (HEXCEL Corporation, Stamford, CT, USA),
- E-glass woven roving/Epidian 601 (R&G Faserverbundwerkstoffe GmbH, Waldenbuch, Germany),
- carbon S&P C-Laminate 150/2000 (S&P Clever Reinforcement Company AG, Seewen, Switzerland).

The material data for the composite materials are collected in Table 2. In all investigated cases, the panels were bonded with the use of S&P Resin 220 Epoxy Adhesive. The thickness of the adhesive layer was about *g*_0_ = 1 mm.

**Figure 3:**
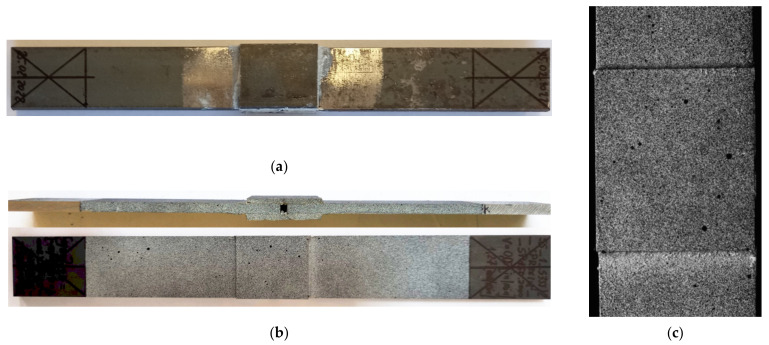
Samples with double-lap joint: (**a**) with metal overlay; (**b**) with composite S&P C-Laminate 150/2000 overlays; (**c**) magnification of speckle pattern on sample with composite overlay.

The first series of performed experimental tests (samples 1–15—Table 3) were focused on the determination of the static and fatigue strength of the double-lap metal–metal and metal–composite adhesive joints. The geometry of the sample with a double-lap joint was assumed with respect to the ASTM D3528 Standard and is presented in Figure 1. The nominal length of the adhesive joint was equal to 13.5 mm and the space between the central part was about 3 mm. The static and fatigue tensile tests were performed for two kinds of overlays—made of steel S355J2+N with thickness *g*_2_ = 4 mm (samples 1–10—i.e., Figure 3a), and S&P C-Laminate 150/2000 with thickness *g*_2_ = 1.4 mm (samples 11–15—i.e., Figure 3b). The detailed list of the samples and applied loading conditions are provided in Table 3, and photographs of samples with double-lap joints are presented in Figure 3.

**Figure 4:**
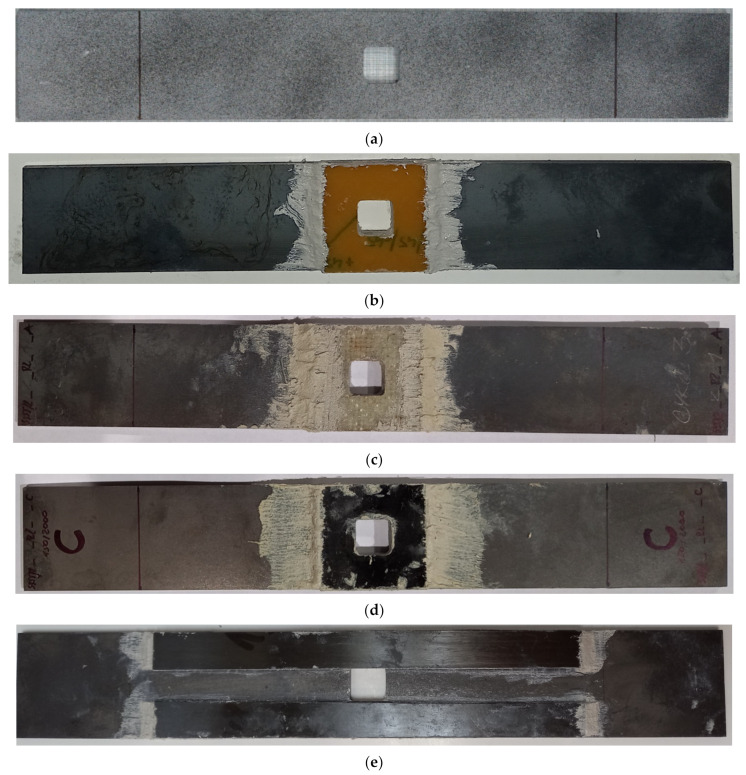
Notched samples with composite overlays: (**a**) sample no. 16 without overlays; (**b**) sample no. 19—Hexcel TVR 380 [+45°/−45°]_4_; (**c**) sample no. 20—E-glass woven roving; (**d**) sample no. 21—S&P C-Laminate 150/2000—45 × 45 mm; (**e**) sample no. 22—S&P C-Laminate 150/2000—15 × 180 mm.

In the second series of the experimental study (samples 16–22—Table 3), the influence of the application of composite overlays on the static and fatigue strength of the notched samples was investigated. The geometry of the non-reinforced steel S355J2+N samples 16 and 17 with the notch are presented in Figure 2a and Figure 4a. The geometries and photographs of the reinforced notched samples are presented in Figure 2b,c and Figure 4b–e, respectively. During the experimental study, two kinds of overlays were used. The first set of overlays (samples 18–21) had a square shape with a size of 45 × 45 mm and had a square hole with rounded corners cut in the centre (Figure 2b). In such a situation, the overlays were made of three different composite materials—Hexcel TVR 380 [+45°/−45°]_4_ (Figure 4b), E-glass woven roving (0°/90°) (Figure 4c), and S&P C-Laminate 150/2000 (0°) (Figure 4d). The second overlay (sample no. 22) had a rectangular shape with 15 × 180 mm size and was bonded on both sides of the notch (Figure 2c). In this case, overlays were made of S&P C-Laminate 150/2000 (0°) (Figure 4e). In all cases, overlays were bonded on both sides of the steel surfaces.

**Figure 5:**
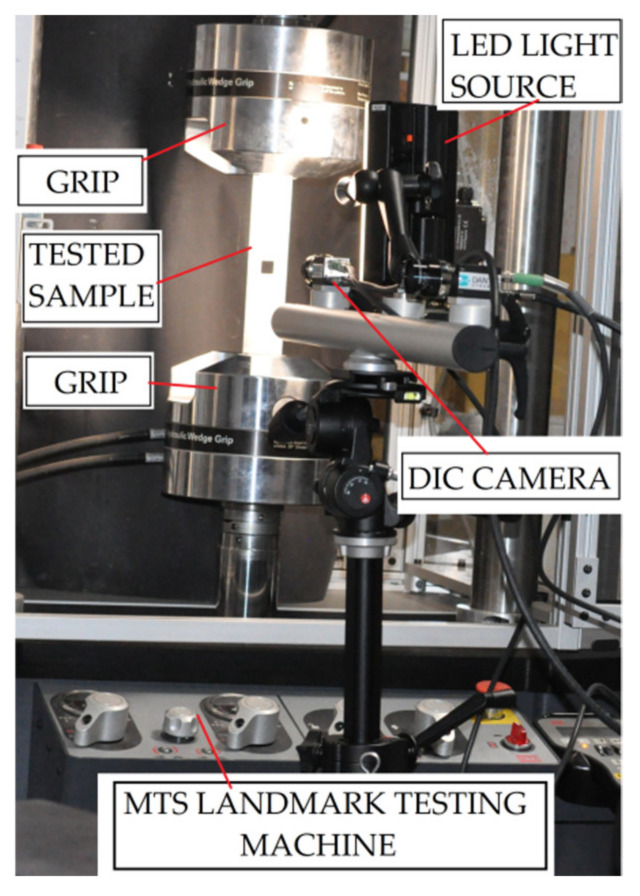
Test setup.

The experimental static and fatigue tests were performed using MTS Landmark 370 servo-hydraulic testing machine with FlexTest 40 controller, MTS 793 System Software and MTS 647.10A grips (the maximal loading—100 kN, all MTS systems corporation, Eden Prairie, MN, USA—Figure 5). All fatigue tests were performed under a constant stress ratio *R* = 0.1 and were conducted using constant amplitude sinusoidal tensile stress cycles with the frequency of 20 Hz. The remaining loading conditions are specified in Table 3.

For the analysis of the surface strains during the static tensile tests, Digital Image Correlation (DIC) was used. The photographs were taken using the high-resolution camera with lens Xenoplan 1.4/23-0902 (Dantec Dynamics A/S, Skovlunde, Denmark, the details of the measurement system are provided in Ref. [14]). During the tests, the samples were illuminated by the use of an external light LED system (Dantec Dynamics A/S, Skovlunde, Denmark). The distance between the camera and the specimen was about 15 cm and photographs were taken with a time interval equal to 2 s. Strain analyses were carried out in GOM Software (GOM GmbH, Braunschweig, Germany) [15,53]. The exemplary speckle pattern on the sample surface—used in the tests—is presented in Figure 3b,c and Figure 4a.

The adhesive joints were built in a few steps. In the first step, the surfaces of adherends were ground in order to remove oxide layers and other surface defects. In the next operation, the surfaces were cleaned with extracted gasoline to remove impurities such as dust and grease. After evaporation of the solvent (about 30 min), sanding was performed in the area of the adhesive joint. Before bonding of adherends with overlays, the surfaces were cleaned with a dry cloth to remove the remaining particles of dust. After bonding, the sample was held in the grips for 24 h and seasoned for a minimum of 7 days.

### 2.2. Theoretical Solution of Double-Lap Joint

The theoretical model of the investigated double-lap joint is based on the shear lag model proposed by Volkersen [39]. The applied solution is based on the given assumptions below:

1. Shear stresses are constant through the thickness of the adhesive joint;
2. Linear and elastic material model;
3. Deformation of adhesive is caused only by shear stress;
4. Deformation of adherends is caused only by tension;
5. Bending moments and peel stresses are neglected.

**Figure 6:**
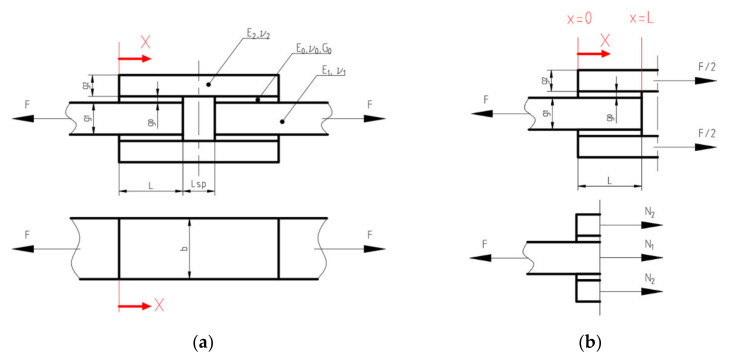
Double-lap joint: (**a**) geometry, dimensions and material parameters; (**b**) internal forces in investigated joint and direction of *x*-axis.

The geometry of the double-lap joint is presented in Figure 6. It is assumed that the joint can be made of different adherend materials determined by thicknesses *g_i_*, Young moduli *E_i_* and Poisson’s ratios ν*_i_*, where *i* = 1 is valid for inner adherend and *i* = 2 refers to outer adherend (overlays). The adhesive material is defined by its thickness *g*_0_ and shear modulus *G*_0_. The length of one side of the joint is equal to *L*, and the space between the inner part is equal to *L_sp_*. The width of both adherends and adhesive joint is equal to *b*, which is also the width of the tested samples.

The distribution of shear stress in the adhesive layer can be calculated along the *x*-axis (Figure 6) from the formula

(1) $$\tau \left(x\right)=\frac{1}{b}\frac{dN_{2}\left(x\right)}{dx}.$$

Shear deformation γ of the adhesive layer can be evaluated in two ways—from shear stress τ and shear modulus *G*_0_ (2), and by taking into account stiffness and forces in both adherends (3):

(2) $$\gamma =\frac{\tau }{G_{0}};\;G_{0}=\frac{E_{0}}{2\left(1+\vartheta_{0}\right)},$$

(3) $$\frac{d\gamma }{dx}=\frac{1}{g_{0}}\left(\frac{N_{2}}{C_{2}}-\frac{N_{1}}{C_{1}}\right);\;C_{1}=E_{1}\cdot b\cdot g_{1};\;\;C_{2}=E_{2}\cdot b\cdot g_{2}.$$

Comparing (3) with differentiated Equation (1) and including dependencies between forces *N*_1_, *N*_2_, and *F*, and after mathematical transformations, the following equation is obtained

(4) $$\frac{d^{2}N_{2}}{dx^{2}}-\omega^{2}N_{2}=-\frac{C_{2}}{C_{1}+2C_{2}}\cdot Q\cdot \omega^{2},$$

where

(5) $$\omega^{2}=\frac{b\cdot G_{0}}{g_{0}}\cdot \frac{C_{1}+2C_{2}}{C_{1}C_{2}}.$$

The solution of the above formulation can be found in the form given below

(6) $$N_{2}=A_{0}+A_{1}\sinh\left(\omega x\right)+A_{2}\cosh\left(\omega x\right),$$

where constant *A*_0_ is equal to

(7) $$A_{0}=\frac{C_{2}}{C_{1}+2C_{2}}\cdot F$$

The constant *A*_1_ and *A*_2_ can be determined from the boundary conditions:

- For *x* = 0: *N*_2_ = 0;
- For *x* = *L*: *N*_2_ = *F*/2;

and they are equal to:

(8) $$A_{1}=\frac{\frac{Q}{2}-A_{0}-A_{2}\cos h\left(\omega \cdot l\right)}{\sinh\left(\omega \cdot l\right)}$$

(9) $$A_{2}=-A_{0}=-Q\frac{C_{2}}{C_{1}+2C_{2}}$$

Finally, the shear stress in the adhesive layer is equal to

(10) $$\tau \left(x\right)=\omega A_{1}\cosh\left(\omega x\right)+\omega A_{2}\sinh\left(\omega x\right).$$

Due to the proposed formulation, the above solution makes possible determination of the shear stress distribution along the joint for different materials and different stiffnesses of adherends.

### 2.3. FEM Models

The analytical solution presented above of double-lap joints can be verified by an approximate numerical solution. Here, the common choice is the finite element method (FEM), which is a well-established tool for engineering analysis of various structural elements, machines, and other objects [18,54,55]. For the detailed computational analysis, the ANSYS software [56] was used.

In general, the FEM analysis can be performed with different levels of approximation, starting from the analysis of the full 3D model of the double-lap joint, which is the most general approach. In the case of the investigated sample, a full 3D model gives the full set of results, including the deformation and stress distributions not only along the horizontal (‘x’ direction, see Figure 6) but also in the remaining orthogonal directions. In order to obtain valuable results, relatively dense element mesh should be applied, particularly in the area of the bonded joint and its vicinity. This, in consequence, leads to time-consuming numerical calculations even in the case of purely elastic analysis. The 3D approach can be simplified into a 2D approach [9] without the loss of the solution’s precision.

**Figure 7:**
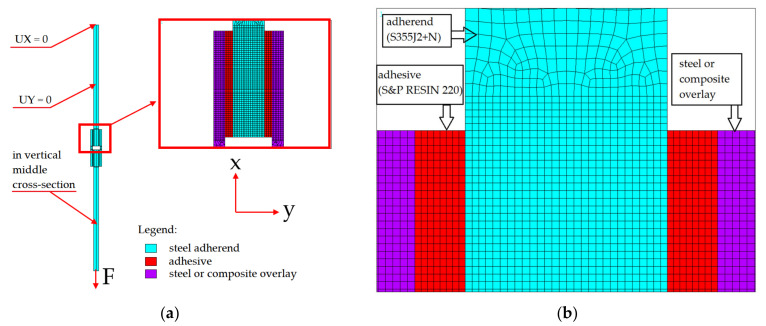
FEM model: (**a**) boundary conditions and part of FEM model with visible half-part of bonded joint and marked materials: light blue—steel adherend, red—adhesive, purple—overlays; (**b**) part of FEM model with finite element discretization.

Due to the symmetry only the centre, a longitudinal cross-section of the sample (Figure 1) with the plane strain state assumption along the symmetry axes can be analysed. Finally, the denser mesh can be used without visible extension of the computation time. In such a case, the results in the middle cross-section part of the model are only accessible and sufficient in the presented study. In the performed 2D analysis particularly, regular dense mesh was used in the adhesively bonded joint and its vicinity. Here the PLANE183 finite element, which is an 8-node structural element with the quadratic approximation of the displacements field, was used. With the use of the KEYOPT command, the plane strain state was declared in the analysis. The parts of the model with moderately dense meshes and boundary conditions used in calculations are shown in Figure 7. To raise the accuracy of the numerical solutions in this mesh, eight elements across the thickness of the adhesive are used with the regular quadrilateral shape. This provides relatively high accuracy of the results. However, due to the high discrepancies between Young’s modulus of adherent and core and overlays, high stress concentrations (peak of the stress) are still observed at both ends of the adhesive area.

## 3. Results

### 3.1. Double-Lap Joints

#### 3.1.1. Static Tensile Test of Double-Lap Joint

**Figure 8:**
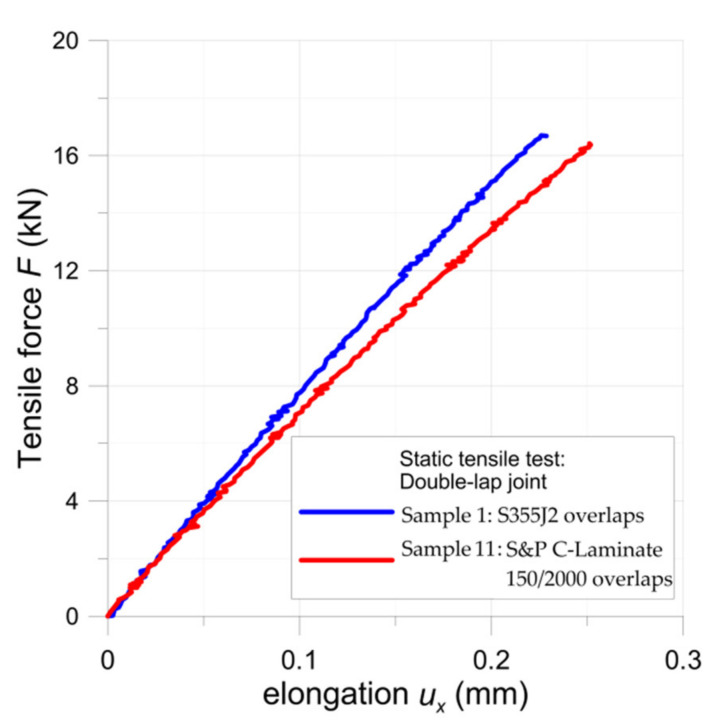
Tensile curves for double-lap joints.

The tensile curves for the samples with double-lap joints with metal S355J2+N (sample no. 1) and S&P C-Laminate 150/2000 (sample no. 11) overlays are given in Figure 8. In both cases, the maximal force was similar (16.7 kN for S355J2+N overlays and 16.4 kN for composite overlays). After failure, detailed measurements of the adhesive joints were carried out and the critical shear stresses were calculated, taking into account the real adhesive area. The estimated average critical shear stress in the adhesive was equal to about 24–25 MPa, which was in good agreement with data provided by the manufacturer.

**Figure 9:**
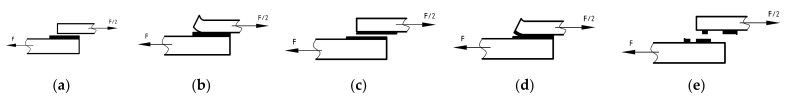
Failure forms of adhesive joints: (**a**) adhesive; (**b**) adhesive caused by peel stress; (**c**) cohesive; (**d**) cohesive caused by peel stress; (**e**) mixed mode.

**Figure 10:**
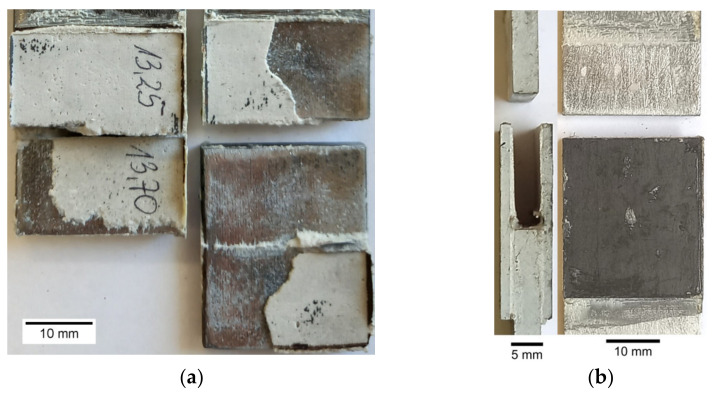
Failure forms of adhesive joints: (**a**) sample no. 1—steel overlays; (**b**) sample no. 11—S&P C-Laminate 150/2000 overlays.

In the general case, the following failure modes can be distinguished in adhesively bonded joints: adhesive failure (Figure 9a,b), cohesion failure of adhesive (Figure 9c,d), mixed-mode (adhesive and cohesive—Figure 9e), or adherend or overlay failure. The failure forms obtained in the experimental static tensile tests are presented in Figure 10. In both cases, the adhesive failure occurred in the samples with the visible detachment from the steel core.

**Figure 11:**
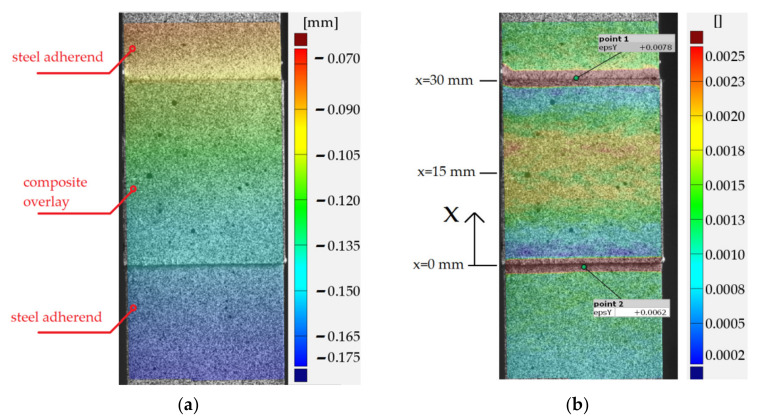
Results from DIC analyses for double-lap joint with S&P C-Laminate 150/2000 overlays: (**a**) vertical displacement *u_x_*; (**b**) major strain ε_x_.

**Figure 12:**
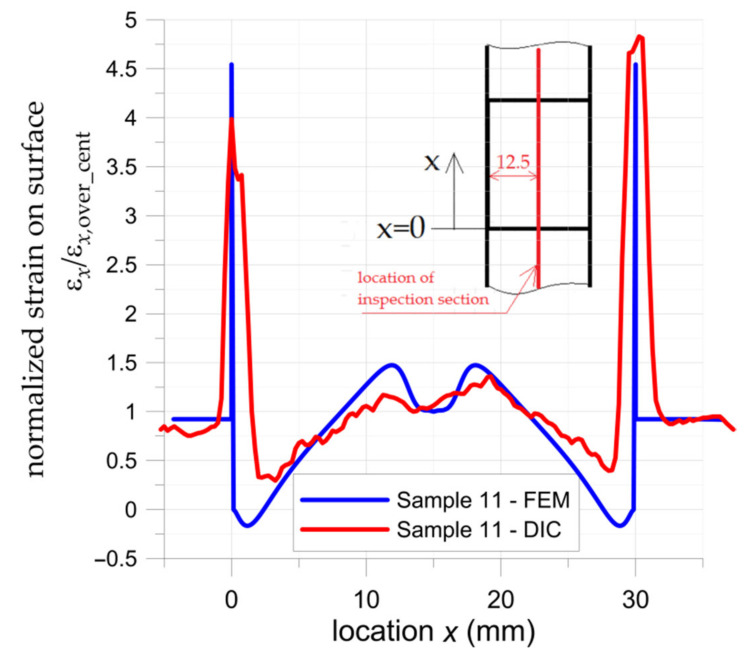
Distribution of normalized surface major strain in middle section of double-lap adhesively joint with S&P C-Laminate 150/2000 overlays—DIC and FEM results.

**Figure 13:**
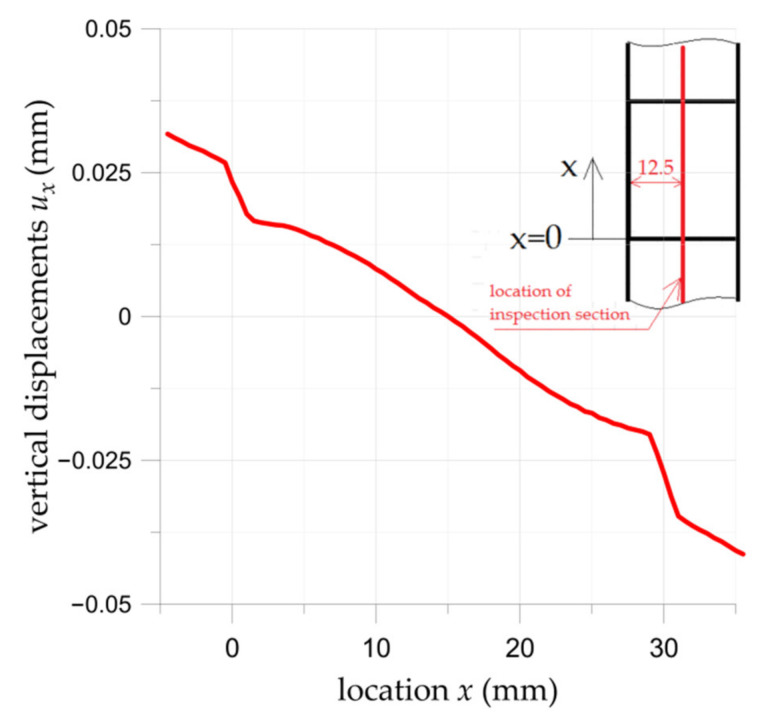
Distribution of surface vertical displacements *u_x_* (DIC) in middle section of double-lap adhesively joint with S&P C-Laminate 150/2000 overlays.

A more detailed study with the application of the DIC was carried out for sample no. 11 with S&P C-Laminate 150/2000 overlays. The results (Figure 11, Figure 12 and Figure 13) are presented for maximal tensile force just before breaking of the adhesive joint. The contour maps of the major displacement *u_x_* and major strain ε*_x_* on parts of the adherends surfaces and overlay determined by DIC are presented in Figure 11a,b, respectively. Obviously, the highest strain is observed in the adhesive layer.

The distribution of the normalized major strain ε*_x_*/ε*_x_*_,over,cent_ in the middle inspection section (parallel to the *x*-axis—see Figure 11b) determined by DIC is compared with the FEM solution in Figure 12. Here, ε*_x_*_,over,cent_ is the strain in the middle cross-section of the overlap (perpendicular to *x*-axis), for *x* = *L* + 0.5 *L_sp_* = 15 mm—more details are given in Figure 6 and Figure 11. The most interesting part is for *x* in the range from 0 to 30, which presents strain on the overlay surface. The ranges *x* > 30 and *x* < 0 show distributions of strain in upper and bottom steel adherends, respectively. Similar to Figure 11 high local peaks appear at the adhesive at the end of the overlay. The shown overlay has been torn off from the upper adherend (*x* = 30 mm) when the highest strain occurred.

The distribution of major strain in the overlay is variable along the bonded joint length and it grows non-linearly from both ends. A slight reduction in surface strain is observed in the central part of the overlay. The above phenomena are observed both in DIC calculations and the FEM solution. Certain differences were observed at the ends of the bonded joint. In the FEM solution, slight compressive effects at the ends of overlays caused by pull-of-bending effects appeared. In the DIC analysis presented in Figure 12 such an effect is not detected due to the influence of significant deformation of the adhesive and the effect of averaging the results with respect to facets size. The more detailed DIC analyses with higher resolutions of facets size in which a small part of overlay was investigated revealed that the strain at the edge was close to 0; however, still compressive effects were not detected.

In Figure 13 the distribution of the *u_x_* displacement in the middle vertical section of the double-lap adhesive joint is presented in the same way as in Figure 12. The rapid changes in displacements are observed at both ends of the overlay (for *x* = 0 and *x* = 30). It is caused by shear deformation γ of the adhesive and leads to the formation of the shear stress in the adhesive (see Equations (2) and (3)).

#### 3.1.2. FEM and Analytical Calculations of Double-Lap Joint

**Figure 14:**
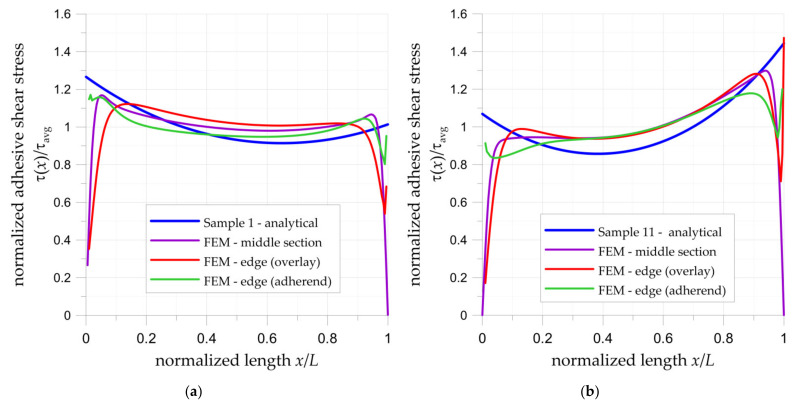
Distribution of normalized adhesive shear stresses in adhesive in double-lap joints calculated by analytical and numerical approaches: (**a**) sample no. 1 with steel overlays; (**b**) sample no. 11 with composite overlays.

The comparison of FEM numerical and analytical results for experimentally tested double-lap joints is given in Figure 14. The calculations were made for maximal tensile forces determined from the tensile tests (Figure 8). Here, the normalized shear strain in the adhesive is calculated as the major shear strain (Equation (10)) divided by the average shear strain. The average shear strain in adhesive was calculated as follows:

(11) $$\tau_{avg}=\frac{F}{2\times b\times L}.$$

In both analytical and FEM analyses, the adhesive thickness was equal to 1 mm. With the use of the FEM, the shear stresses were determined in planes: (1)—on the edge of the adhesive and adherend surface, (2)—in the middle section of adhesive, (3)—on the edge of the adhesive and overlay surface. The largest differences between numerical and analytical results are observed at the ends of the adhesive joint (Figure 14). In the analytical solution, the highest stresses appear at both ends of the joint. On the other hand, in the FEM solutions, the distribution of shear stress strongly depends on the location of the section plane and in some cases, a substantial decrease in shear stress is visible. In both investigated examples, the highest drop is observed on both sides in the middle section of adhesive and on one side of the surface of the joint with overlay. On the surface of the joint with adherend, no decrease or only a slight reduction in shear stresses has been observed. It can be observed that in both cases the maximal stresses at the ends calculated by FEM are comparable with values obtained from the analytical solution. However, it should be noted that in the FEM, there is a strong geometrical notch at the ends of overlays, which disturbs obtained results.

The lowest shear stresses appear in the central part of the joint. Excluding both ends, the FEM and analytical results are in good agreement, both in relation to the trend and values of stresses. Non-symmetrical distributions of the stresses through the bonded joint length are caused by unbalanced stiffnesses of the adherends and overlays. Larger differences between the maximal stresses occurred in the sample with composite overlays.

The determined stress concentrations at the ends are potential points of the failure initiation of the adhesive joint. In the case of the double-lap joint with steel overlays, the maximal stresses occur at endpoints of the joint (*x* = 0). In the case of the double-lap joint with composite overlays, the maximal stresses occur at the centre of the joint at the point at which the initial gap starts (*x* = *L*).

**Figure 15:**
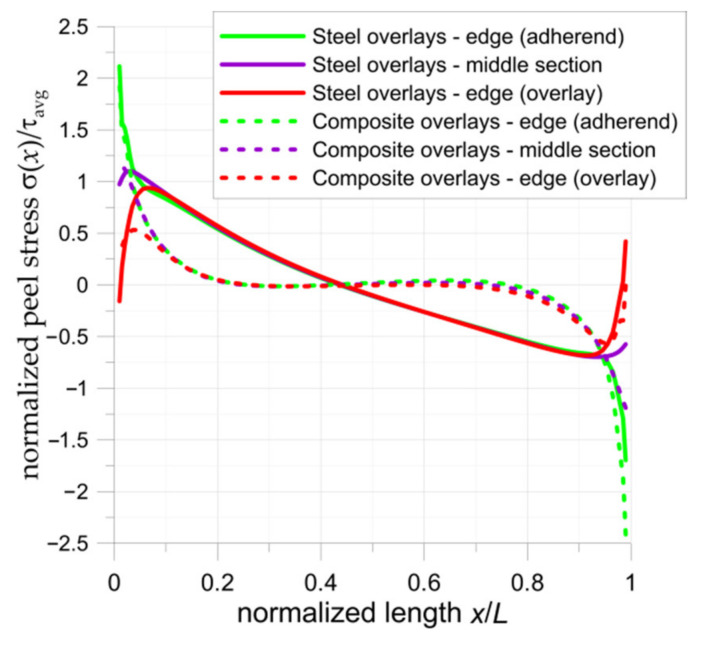
Distribution of normalized peel stresses in samples with double-lap joints.

The determined adhesive shear stresses are the main cause of joint failure; however, it should be noted that the strength of the adhesive joint may be decreased by the occurrence of peel stresses. It is observed that tensile peel stresses have a negative influence on the durability of the adhesive joint and reduce the allowable shear stress in the adhesive. The highest peel stresses occur at both ends of the adhesive due to the bending moments (see FEM solution given in Figure 15). In the case of a double-lap joint, the most unfavourable cases are in the sections at the end of overlays (*x*/*L* = 0), where peel stresses achieve the highest tensile levels. At the ends of the adherends in the middle part of the bonded joint (*x*/*L* = 1) generally compressive peel stresses appear. More information about peel stresses can be found in [28,30,34]. In both investigated cases, the highest tensile effects are observed on the edge of the adherend. It should be also noted that, on the edge of overlay (*x*/*L* = 1), slight tensile peel stresses also appeared. In the case of the composite overlays, the peel stresses appear only at both ends; however, in the case of steel overlays, a smoother and almost linear change (from tensile to compressive) in peel stresses is observed.

#### 3.1.3. Fatigue Tensile Tests of Double-Lap Joint

**Figure 16:**
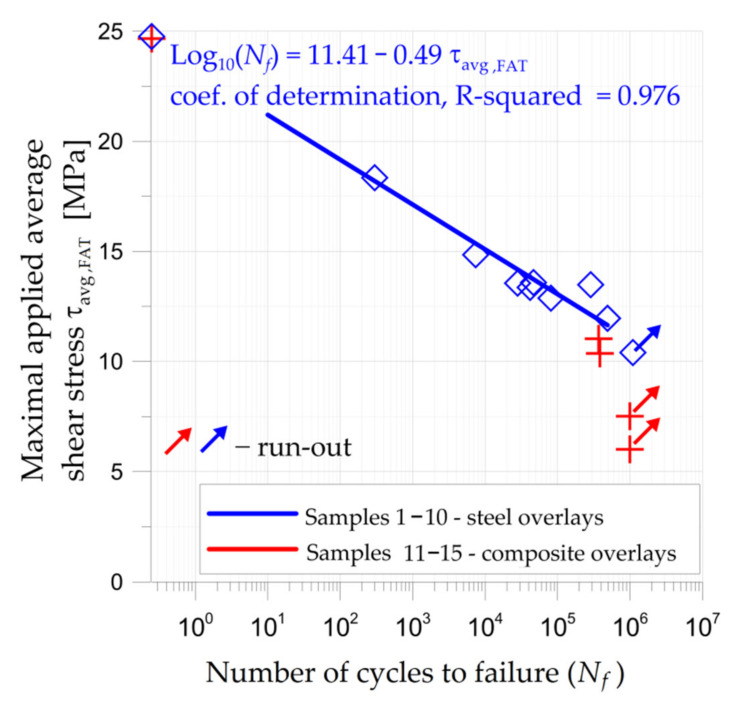
Results of fatigue tests for samples with double-lap joints.

The results of the fatigue tests are given in Figure 16. Such fatigue tests were carried out for nine samples (2–10) with steel overlays (the maximal applied stresses varied between 10.40–18.35 MPa, while the maximal average adhesive shear stress was equal to 24.76 MPa) and four samples 12–15 with composite S&P C-Laminate 150/2000 overlays (the maximal applied stresses varied between 6.01–11.03 MPa, while the maximal average adhesive shear stress was equal to 24.67 MPa). The maximal average adhesive shear stresses were determined from the tensile tests (see Figure 8 and Section 3.1.1). The studies were focused on the determination of the fatigue strength limits of the adhesive joints for a number of cycles within the range 3 × 10^5^–10^6^ cycles.

The first fatigue tests were carried out for the samples with steel overlays (samples 2–10). The expected number of fatigue cycles (above 3 × 10^5^ cycles) was achieved for fatigue stresses below 12 MPa. The fatigue life of the specimen subjected to the maximal fatigue stress 11.96 MPa (corresponding to 48.3% of static tensile strength) was 489,873 cycles. The sample subjected to the maximal fatigue stress of 10.4 MPa (42% of static tensile strength) did not fail at 1.1 × 10^6^ cycles.

Based on the results of fatigue tests for samples with steel overlays, experimental tests for samples 12–15 with composite S&P C-Laminate 150/2000 overlays were conducted. In such a case, the main aim of these experimental studies was to confirm the fatigue limit for 3 × 10^5^—10^6^ cycles. The samples subjected to the maximal fatigue stress 7.51 MPa and 6.01 MPa (30% and 24% of static tensile strength, respectively) did not fail at 1.0 million cycles. For higher fatigue loads (maximal fatigue stress 10.36 MPa and 11.03 MPa) the fatigue life was reduced to 3.83 × 10^5^ cycles and 3.71 × 10^5^ cycles, respectively. Such loadings correspond to 42% and 45% of static tensile strength of samples with composite S&P C-Laminate 150/2000 overlays.

**Figure 17:**
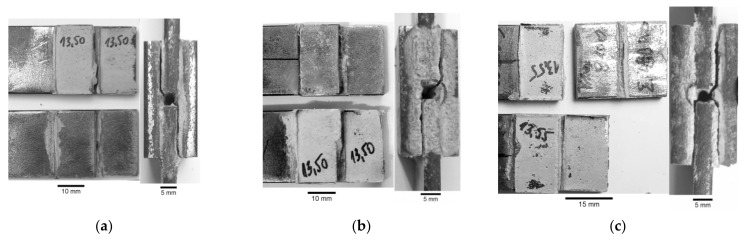
Failure modes of samples with steel overlays with visible steel/adhesive interfacial failure: (**a**) τ_avg,FAT_ = 18.35 MPa; (**b**) τ_avg,FAT_ = 13.48 MPa; (**c**) τ_avg,FAT_ = 11.96 MPa.

**Figure 18:**
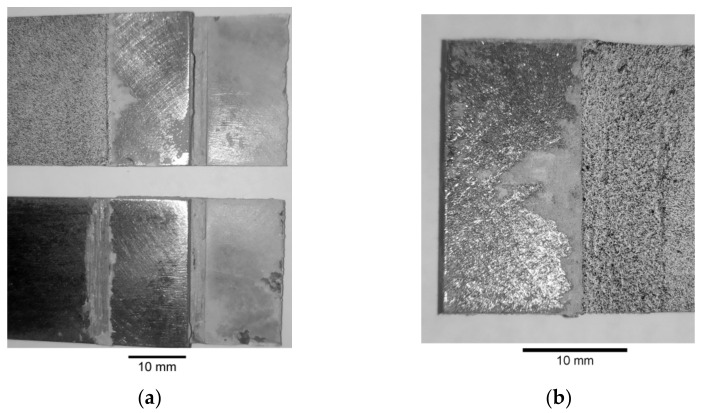
Failure modes of samples with composite overlays with visible steel/adhesive interfacial failure: (**a**) τ_avg,FAT_ = 11.03 MPa; (**b**) τ_avg,FAT_ = 10.36 MPa.

In both cases, the typical failure forms of samples can be identified from the photographs given in Figure 17 (samples with steel overlays) and in Figure 18 (samples with composite S&P C-Laminate 150/2000 overlays). In all cases, the failure was caused by the interface debonding between adhesive and steel parts. Only in a few cases, small zones of cohesive rupture were observed (Figure 18b).

It should be noted that the S–N curve for sample with steel overlays (Figure 16) was determined from a small number of fatigue tests. The exact determination of S–N curves is a complicated issue and requires numerous extensive fatigue tests. Because of this, such results can be used for coarse estimation of the fatigue limit or life of the adhesive layer. The conclusion of the performed tests (considering keeping high fatigue failure at the level not less than 3 × 10^5^ cycles) was that the approximate 55%–60% reduction of the fatigue strength with respect to the tensile strength was obtained. In the presented study such results were used for the determination and verification of the size of the overlays for notched metal samples discussed in Section 3.2.

### 3.2. Notched Steel Samples Reinforced by Composite Overlays

#### 3.2.1. Static Tensile Test

**Figure 19:**
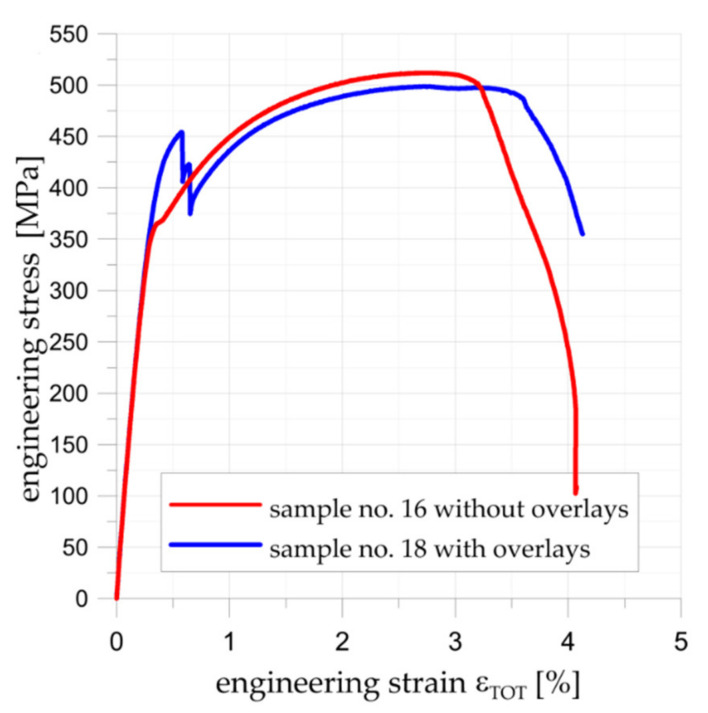
Comparison of tensile curves for samples without (no. 16) and with composite overlays (no. 18).

**Figure 20:**
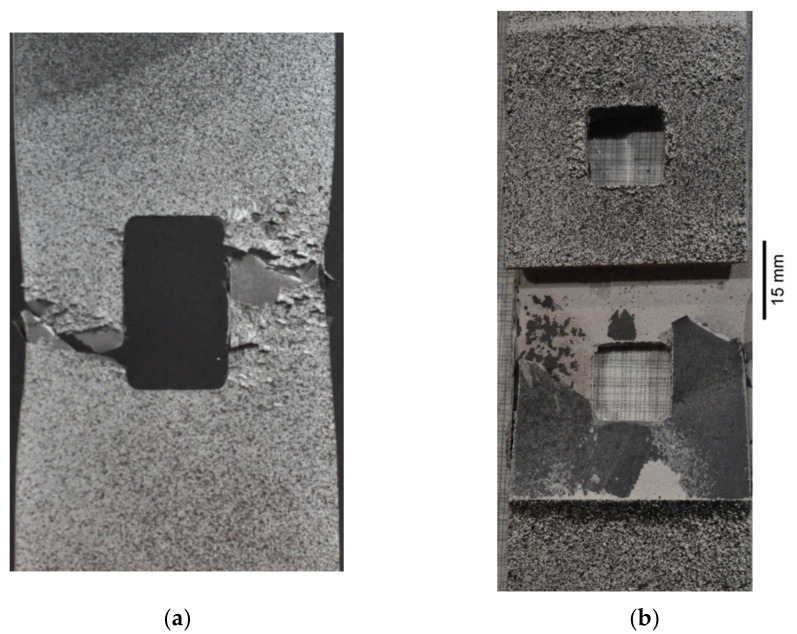
Failure form of notched samples: (**a**) no. 16 without overlays; (**b**) no. 18 with composite Hexcel TVR 380 [+45°/−45°]_4_ overlays. Samples were covered by speckle patterns for DIC analyses.

**Figure 21:**
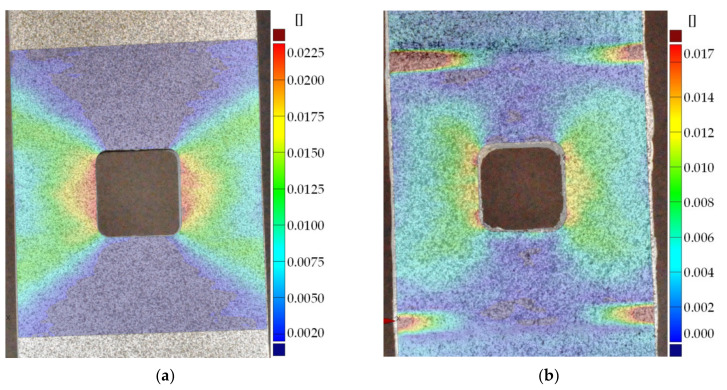
Surface strains determined from DIC analyses (total strain ε_TOT_ = 0.48%): (**a**) sample no. 16 without overlays; (**b**) sample no. 18 with composite Hexcel TVR 380 [+45°/−45°]_4_ overlays.

Static tensile tests were carried out for unreinforced sample no. 16 and sample with composite overlays (no. 18). A comparison of stress–strain curves for both samples is given in Figure 19. The application of the rectangular 45 × 45 mm overlays increased the load-carrying capacity in the range of elastic response. The maximal loading for the reinforced sample was increased by about 25% in comparison with the yield response of the unreinforced sample no. 16. Exceeding this limit leads to steel/adhesive interfacial failure (Figure 20b). Failure forms of both samples are presented in Figure 20. For a more detailed analysis of the behaviour and failure mechanism of the reinforced sample, the DIC studies were carried out. The presented surface strain distributions (Figure 21) are determined for the tensile load at the level corresponding to the total strain ε_TOT_ = 0.48% (see Figure 19). It can be seen that on the composite overlay the zone with high major strain has been increased (Figure 21b) in comparison with the unreinforced sample (Figure 21a). This confirms the possibility of increasing the load-carrying capacity on a notched object by reinforcing overlays. The highest strains are observed in the adhesive in the corners of the overlays (Figure 21b). Such stress concentrations caused by shear stress and peel stress in adhesive finally lead to steel/adhesive interfacial failure. A more detailed study of this problem can be found in [9].

#### 3.2.2. Fatigue Tensile Tests of Notched Samples Reinforced by Composite Overlays

Fatigue tests were performed under the same loading conditions. The stress ratio *R* = 0.1 and the maximal loading *F_max_* = 44.1 kN were applied during the fatigue tests. The experimental tests were performed for non-reinforced notched sample no. 17 (geometry in Figure 2a) and notched samples no. 19–22 with composite overlays (geometries in Figure 2b,c). The main aim of this experimental study was a preliminary assessment of the possibilities of increasing the fatigue life by the application of composite overlays. The fatigue life was determined as the rupture of the adherend (core) part of the sample. The initial size of the overlays (Figure 2b) was assumed on the basis of the static tensile test (Figure 19) and fatigue tests carried out for samples with double-lap joints (Section 3.1.3).

**Figure 22:**
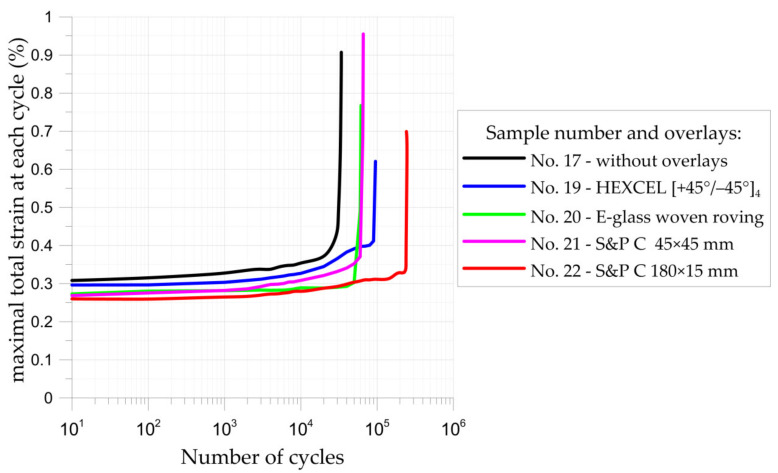
Maximal total strain vs. number of cycles curves for notched samples.

**Figure 23:**
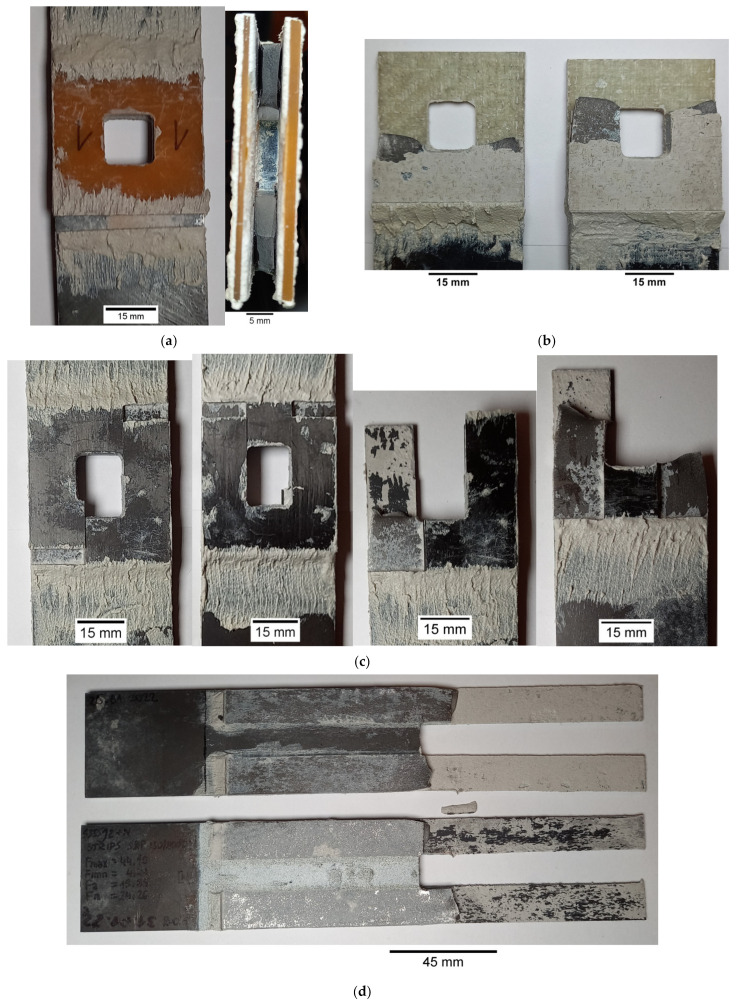
Failure modes of samples with composite overlays: (**a**) no. 19—Hexcel TVR 380 [+45°/−45°]_4_; (**b**) no. 20—E-glass woven roving; (**c**) no. 21—S&P C-Laminate 150/2000—45 × 45 mm; (**d**) no. 22—S&P C-Laminate 150/2000—15 × 180 mm.

Neglecting the effect of the stress concentrations at the notches, the maximal applied fatigue loads correspond to tensile stress 367.5 MPa in the weakest cross-section of not reinforced sample no. 17 (see Figure 19). Taking into account the stress concentration factor, which achieves in the investigated case value *K_t_* = 2.5 [9,15], it can be concluded that the maximal applied stresses at the notches were much higher than the yield limit of the steel core. The existence of such high stresses (theoretical stress at the notch is 919 MPa, while the minimum guaranteed yield limit is 355 MPa; the determined yield limit from the tensile test was 372 MPa [9]) resulted in the short fatigue life of non-reinforced sample no. 17, namely *N_f_* = 34,303 cycles to rupture (see Figure 22 and Table 4). Failure forms of the investigated samples are presented in Figure 23.

The application of the square composite overlays with dimensions 45 × 45 mm (samples no. 19–21) increased the fatigue life 1.8–2.7 times in comparison with non-reinforced sample no. 17. In these cases, the fatigue strength was limited by the insufficient area of adhesive connection. The permissible shear stresses in adhesive were exceeded due to the plastic deformation of the adherend and high-stress concentrations around the notches (see Figure 21b). It should be noted that admissible fatigue adhesive shear stress is significantly reduced in comparison with static strength (usually about 50%—see Section 3.1.3 and Figure 16, but sometimes it can be reduced even by 80%).

**Figure 24:**
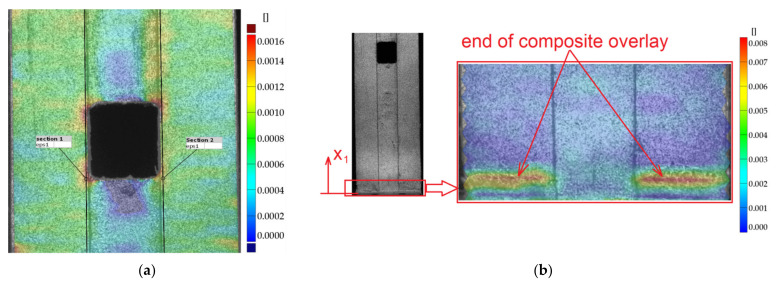
Distribution of surface strain (DIC) for sample no. 22 for maximal load 44.1 kN at 0.25 cycle: (**a**) around notch; (**b**) strain concentrations in adhesive at ends of overlays.

**Figure 25:**
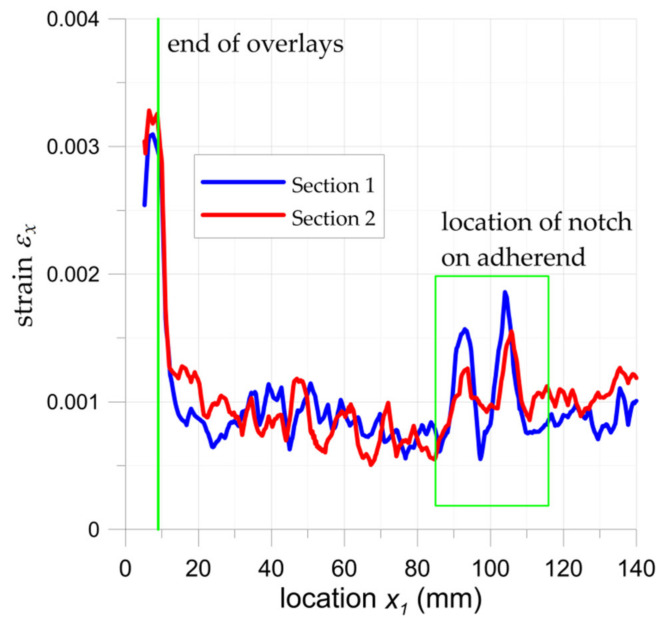
Distribution of surface strain in two sections (from DIC analysis) for sample no. 22 for maximal load 44.1 kN at 0.25 cycle in Section 1 and Section 2 (both sections are marked in Figure 24).

The highest increase in the fatigue life (7.1 times) was achieved for the sample with overlays in the form of stripes with dimensions 180 × 15 mm (sample no. 22). The application of longer overlays (180 mm instead of 45 mm in length) balanced adhesive shear stresses across the entire bonded joint (Figure 24a and Figure 25). Good relief of the notches at the adherend was also observed (see local concentrations for *x*_1_ = 85–115 mm on the outer surface of the overlay—Figure 25, which indicates the proper load transfer from the core). During the fatigue test, the first damage in the adhesive layer was caused by peel stresses at both ends of the overlays (see Figure 24b). Further degradation results in steel/adhesive interfacial failure and further cracks forming and propagating in adherend.

## 4. Discussion

The performed static analyses (theoretical, numerical and experimental with the use of DIC system) revealed significant improvement of the structure strength when applying reinforcing overlays. However, such improvement of the static strength is limited by the appearance of the peel stresses at the end adhesive joints. The performed experimental studies allow for assessment of loading conditions applied in the fatigue tests. The comparison of the static and fatigue strengths of the samples with double-lap joints show that the fatigue strength is strongly reduced in comparison with the static tensile strength of the adhesive (more than 50%).

The static and fatigue strength of the adhesive bonded joints depends on different factors, such as bonding area, surface conditions, type of adhesive and adherends, geometry (thicknesses), material properties, residual stresses, the shape of a spew fillet at the ends of the overlap, effect of peel stresses, etc. [35]. The assurance of stable values of the above factors is quite difficult and fatigue strength of the adhesive-bonded joint may reveal higher scatter than metal samples. Optimization of the adhesive joints can be accomplished with the use of FEM or theoretical models. For typical joints such as single-lap or balanced double-lap joints, the analytical solution can be found in the literature [28,37,39,40,41,42,43,44,45]. The analytical solution for the non-balanced double-lap joint investigated in the paper was derived in Section 2.2 and verified by FEM in Section 3.1.1. The comparison of both solutions for adhesive shear stress shows good agreement with both approaches. The analytical approach offers possibilities for optimal shaping of the designed joint. In this way, the thickness, length, and width of the overlays and the adhesive layer thickness with respect to the material properties can be established. However, this approach does not allow the study of the influence of the shape of the overlays, such as variable geometry along its length, the shape and dimensions of ending edges of adherends and adhesive fillets on the durability of samples [10,35]. This inconvenience may be compensated by the use of FEM. Such analysis is much more versatile than the common analytical approach. It provides more freedom in the choice of the geometrical parameters defining the bonded joints. This opens the space for numerical optimization of adhesive joints and results in optimal sets defining the joint geometry.

The influence of the material properties and thickness of the overlay was discussed in the paper by the use of analytical and numerical approaches. It was observed that the distribution of adhesive shear and peel stresses in the non-balanced joint is more unfavourable than in the balanced one [28]. The performed experimental tests confirmed that higher concentrations of stresses at the ends of joints reduce the fatigue strength of a structure.

The performed experimental study for notched samples shows that the application of the overlays may significantly increase the fatigue life of notched structures. It has been shown that even the use of the small overlays (the external dimension three times larger than the notch size) increases fatigue life by about 180–270% in comparison with the unreinforced sample. Such a slight increase in durability is caused by high adhesive shear stresses in the notched area (reduction of *K_t_*—see Table 5).

Based on the static tensile test for the same geometry (Figure 19), the critical static shear stress in adhesive was estimated taking into account the drop of the tensile load at the moment of the overlay debonding. In such a situation (for the static tensile load of 54.48 kN) the average critical adhesive shear stress was equal to about 6.5 MPa. This level of stress corresponds to the high fatigue life (at least 1 million cycles) of specimens with double-lap joints (Section 3.1.3). However, taking into account the stress concentration around the notches (*K_t_* = 2.183—Table 5), the maximal adhesive shear stress may achieve 14 MPa (for the maximal load of 54.48 kN) which corresponds to the low fatigue regime. The experimental fatigue tests were carried out for lower loadings; however, the concentrations of the shear stresses in the adhesive were still at a high level.

The highest increase in fatigue life (by 710%) was accomplished by a triple increase in adhesive area. In this case, failure of the adhesive joint was caused by the existence of tensile peel stresses at the overlay ends. The reduction of such peel stresses seems to be a key issue for further research (i.e., by chamfering of the overlays). It should be emphasized that the obtained improvement of the fatigue strength was obtained under high tensile loadings (the tensile stress in the not reinforced sample was at the yield limit, and the maximal stress at the notch exceeded the yield limit by about 2.47 times).

A summary of the obtained results is given in Table 5. The stress concentration factors were calculated by means of FEM (more details about FEM models are given in [9]) for particular geometries (overlay material and shape) with the same notch geometry in the steel adherend part and the same loading conditions (see Table 3). The results show the benefits from the use of the reinforcing overlays applied in the vicinity of a notch. The maximal stress reduction (minimum *K_t_*) is observed after the application of two long reinforcing paths (size 180 × 15 mm) made from composite material.

## 5. Conclusions

The static and fatigue studies of the adhesive bonded joints are presented and discussed in the paper. On the basis of the performed analytical, numerical, and experimental investigations, the following conclusions can be drawn:

− The static tensile strengths of double-lap joints with the same adhesive area were similar for samples with overlays made of different materials and thicknesses; however, the fatigue strength of adhesive in double-lap joint strongly depends on the stiffness of the adherend–overlays arrangement,
− The analytical formulation used for the calculations of the shear stresses in double-lap joints shows good agreement with the numerical solution,
− The application of the DIC system reveals good convergence with the FEM solution and enables the determination of the strain concentrations at the notches,
− The application of the overlays increases load-carrying capacity under static tensile loading conditions,
− The fatigue strength of the notched samples can be significantly increased by the application of the overlays. However, the weakest point of such a joint is the steel/adhesive connection,
− The fatigue strength of the adhesive joint can be increased by increasing the adhesively bonded area. However, additional technological treatments (i.e., chamfering of the overlays) are necessary to reduce peel stresses at the ends of the joint.

## Figures and Tables

**Table 1:** Chemical composition of S355J2+N steel.

Chemical Components of S355J2+N Steel (in Weight %)									
Material	C	Si	Mn	P	S	Cu	Al	Cr	Fe
S355J2+N (tested material) [9]	0.19	0.20	0.99	0.012	0.01	0.03	0.04	0.02	res.
S355J2, Standards [49]	0.20–0.22	0.55	1.60	0.025	0.025	0.55	-	-	res.

**Table 2:** Mechanical material properties.

Steel							
Material	*E* [GPa]		ν		*Y_eH_* [MPa]		*f_u_* [MPa]
S355J2+N	210		0.3		Min 355		470–630
**Composites**							
**Material**	***E*** **_1_** **[** **GPa]**	***E*** **_2_** **[GPa]**	***G*** **_12_** **[** **GPa]**	***G*** **_23_** **[GPa]**	**ν_1_**	**ν_2_**	***f_u_*** **[** **MPa]**
HEXCEL TVR 380 M12/26%/R-glass/epoxy [+45°/−45°]_4_ [9,12]	46.43	14.92	5.23	9.15	0.269	0.3	141.8
E-glass woven roving/Epidian 601 [50]	16.8	16.8	3.4	3.4	0.14	0.14	220
S&P C-Laminate 150/2000 [51]	165	10	5	5	0.3	0.3	2800
**Adhesive**							
**Material**	***E*** **[** **GPa]**	**ν**		**τ_adh_** **[MPa]**	**Adhesion steel to steel** **(tensile strength) [MPa]**		
S&P Resin 220 Epoxy Adhesive [52]	7.1	0.35		26	14		

**Table 3:** Details of experimental research program.

Sample Number *i*	Geometry	Type of Tension Load	Overlay Material	Overlay Thickness *g*_2_ in mm	Loading Conditions ^1^
First series of experimental tests—double lap-joint (DLJ) samples					
1	Figure 1	Static	S355J2+N	4	*v* = 0.5 mm/min
2–10	Figure 1	Fatigue	S355J2+N	4	*R* = 0.1, 9 samples tested, τ_avg,FAT =_ 18.4, 14.9, 13.6, 13.6, 13.5, 13.4, 12.9, 12.0, 10.4 MPa
11	Figure 1	Static	S&P C-Laminate 150/2000	1.4	*v* = 0.5 mm/min
12–15	Figure 1	Fatigue	S&P C-Laminate 150/2000	1.4	*R* = 0.1, 4 samples tested, τ_avg,FAT =_ 11.0, 10.4, 7.5, 6.0 MPa
**Second series of experimental tests—notched steel samples reinforced by composite overlays**					
16	Figure 2a	Static	without	-	*v* = 0.5 mm/min
17	Figure 2a	Fatigue	without	-	*F_max_* = 44.1 kN, *R* = 0.1
18	Figure 2b	Static	HEXCEL TVR 380 [+45°/−45°]_4_	2.1	*v* = 0.5 mm/min
19	Figure 2b	Fatigue	HEXCEL TVR 380 [+45°/−45°]_4_	2.1	*F_max_* = 44.1 kN, *R* = 0.1
20	Figure 2b	Fatigue	E-glass woven roving	2.1	*F_max_* = 44.1 kN, *R* = 0.1
21	Figure 2b	Fatigue	S&P C-Laminate 150/2000	1.4	*F_max_* = 44.1 kN, *R* = 0.1
22	Figure 2c	Fatigue	S&P C-Laminate 150/2000	1.4	*F_max_* = 44.1 kN, *R* = 0.1

^1^ *v*—tensile speed in mm/min, τ_avg,FAT_—maximal applied value of average adhesive shear stress during fatigue test in MPa, *F_max_*—maximal value of the applied fatigue force in kN.

**Table 4:** Results of experimental fatigue tests of notched metal samples; maximal tensile force *F_max_* = 44.1 kN, stress ratio *R* = 0.1.

Sample Number *i*	Overlay Material	Fatigue Life *N_f_* (in Cycles)	δinci=NfiNf17	Failure Form
17	without	34,303	1	-
19	HEXCEL TVR 380 [+45°/−45°]_4_	95,377	2.7	Slight fibre/matrix debonding around notches and adhesive failure of bonded joint—Figure 23a
20	E-glass woven roving	61,910	1.8	Adhesive failure of bonded joint—Figure 23b
21	S&P C-Laminate 150/2000	66,250	1.9	Overlay failure and adhesive failure of bonded joint—Figure 23c
22	S&P C-Laminate 150/2000	242,500	7.1	Adhesive failure of bonded joint—Figure 23d

**Table 5:** Stress concentration factor at notch in adherend, θ—fibre angle orientation in layers with respect to tension direction.

Overlay Dimensions in (mm)	*K_t_* (−)	θ	Corresponding Sample Number *i*
**Without Overlay**			
**-**	2.508	-	17
**Rectangular Patch**			
Size (45 × 45)	2.183	[+45°/−45°]_4_	19
Size (45 × 45)	2.014	[0°]_8_	21
Size (180 × 15)	1.366	[0°]_8_	22

## Data Availability

The data presented in this study are available on request from the corresponding author.

## Nomenclature

**Abbreviations**	
CFRP	carbon-fibre-reinforced plastics,
DIC	digital image correlation,
DLJ	double-lap joint,
FEM	finite element method,
GFRP	glass-fibre-reinforced plastics,
**Variables**	
*A*_0_, *A*_1_, *A*_2_	constants of theoretical solution of DLJ,
*b*	width of sample,
*C*_1_, *C*_2_	stiffness of adherend and overlap, respectively,
*E*	Young stiffness modulus,
*F*	applied tensile force,
*F_max_*	maximal applied value of tensile force during fatigue test,
*f_u_*	ultimate tensile strength,
*v*	tensile speed,
*G*	shear modulus,
*g*_0_	adhesive thickness,
*g*_1_	adherend thickness,
*g*_2_	overlap thickness,
*i*	number of samples
*K_t_*	stress concentration factor,
*L*	length of one side of DLJ joint,
*L_sp_*	space between inner parts of DLJ,
*N*_1_, *N*_2_	forces in adherend and overlaps in particular cross-section, respectively,
*N_f_*	number of cycles to failure,
*R*	stress ratio,
*u_x_*	elongation of sample in tension direction,
*Y_eH_*	Yield limit,
ε_TOT_	total strain of sample in tension direction,
ε_x_	major strain in tension direction,
ε*_x_*_,over,cent_	strain in middle cross-section of overlap,
ν	Poisson’s ratio,
*δ_inc_*	ratio of increase of fatigue life of reinforced sample in relation to not reinforced one,
θ	fibre angle orientation in layers with respect to tension direction,
σ	peel stress in adhesive,
τ	shear stress in adhesive,
τ_adh_	adhesive shear strength,
τ_avg_	average shear stress in adhesive,
τ_avg,FAT_	maximal applied value of average adhesive shear stress during fatigue test,
γ	shear deformation.
